# Deep Eutectic Solvents for Efficient Drug Solvation: Optimizing Composition and Ratio for Solubility of β-Cyclodextrin

**DOI:** 10.3390/pharmaceutics15051462

**Published:** 2023-05-11

**Authors:** Ilan Shumilin, Ahmad Tanbuz, Daniel Harries

**Affiliations:** 1 Institute of Chemistry, The Hebrew University, Jerusalem 9190401, Israel; ilan.shumilin@mail.huji.ac.il (I.S.);; 2 The Fritz Haber Research Center, The Hebrew University, Jerusalem 9190401, Israel; 3 The Harvey M. Krueger Family Center for Nanoscience and Nanotechnology, Edmond J. Safra Campus, The Hebrew University, Jerusalem 9190401, Israel

**Keywords:** solubility, chemical potential, activity, drug, deep eutectic solvent, hydration

## Abstract

Deep eutectic solvents (DESs) show promise in pharmaceutical applications, most prominently as excellent solubilizers. Yet, because DES are complex multi-component mixtures, it is challenging to dissect the contribution of each component to solvation. Moreover, deviations from the eutectic concentration lead to phase separation of the DES, making it impractical to vary the ratios of components to potentially improve solvation. Water addition alleviates this limitation as it significantly decreases the melting temperature and stabilizes the DES single-phase region. Here, we follow the solubility of β-cyclodextrin (β-CD) in DES formed by the eutectic 2:1 mole ratio of urea and choline chloride (CC). Upon water addition to DES, we find that at almost all hydration levels, the highest β-CD solubility is achieved at DES compositions that are shifted from the 2:1 ratio. At higher urea to CC ratios, due to the limited solubility of urea, the optimum composition allowing the highest β-CD solubility is reached at the DES solubility limit. For mixtures with higher CC concentration, the composition allowing optimal solvation varies with hydration. For example, β-CD solubility at 40 wt% water is enhanced by a factor of 1.5 for a 1:2 urea to CC mole ratio compared with the 2:1 eutectic ratio. We further develop a methodology allowing us to link the preferential accumulation of urea and CC in the vicinity of β-CD to its increased solubility. The methodology we present here allows a dissection of solute interactions with DES components that is crucial for rationally developing improved drug and excipient formulations.

## 1. Introduction

Ranging from design and synthesis of drugs to their solubilization and delivery, deep eutectic solvents (DESs) have shown striking potential as effective green and inexpensive solvents for various pharmaceutical applications [1,2,3]. DESs are formed by mixing Lewis or Bronsted acids and bases at precise concentration ratios, and these typically contain a variety of charged ionic species as well as neutral components [1,4]. The eutectic point is characterized by a low melting temperature, so that DESs are liquid at or near room temperature, in contrast to the comprising components that are solid [5,6]. Because they typically constitute large concentrations of salt, DESs can also be considered as an ionic liquid (IL) [7,8]. In both ILs and DESs, competing interactions that include hydrogen bonding, steric, and coulomb forces, subsequently lead to molecular packing frustrations that significantly lower the melting temperature of the mixture [1,9,10,11,12,13].

The complex structure of DESs makes it challenging to improve their solvation capacity. One reason is that changing the DES composition from the eutectic ratio at temperatures not much higher than the eutectic temperature leads to precipitation of one or more of its components [5,6]. This typically allows only a narrow range of ratios at which a single liquid phase is still maintained. Using a higher temperature to extend the range of ratios is often impractical due to the limited thermal stability of drugs and the limited temperature range of their administration [14,15,16]. Another reason is that the inability to vary solution composition makes it difficult to identify which component contributes most to solvation, and even more so to identify whether the DES components act synergistically to improve solvation in concert. 

Water addition to DES has been shown to significantly lower the melting point, allowing the DES components to remain liquid also at concentrations away from the eutectic ratio [9,17,18]. Adding even small amounts of water has proven beneficial not only for optimizing solubility but also to significantly reduce viscosity, allowing easier solute dissolution [19]. Water was found to change the molecular organization and structuring of DES components on the nanoscale, potentially impacting its solvation properties towards different solutes [9,20,21,22]. In addition to the advantages of water as an additional DES component, this also presents further challenges towards resolving the solvation mechanism. In fact, very few studies have explored complex solvation in mixtures with four or more components [23,24,25,26,27,28].

A variety of experimental methods, including X-ray or neutron scattering, nuclear magnetic resonance, and infrared spectroscopy, have been previously employed to probe interactions between DES components and solubilized solutes [9,29,30,31,32,33,34]. These methods not only allow the determination of specific interactions in complex solutions such as hydrated DESs, but also facilitate the detection of host–guest complexation of macrocycle excipients with drug compounds, a property exploited in many drug formulations [35,36,37]. However, these methods are limited, as they follow structural aspects of interactions, which are not easily linked to thermodynamic properties. We focus here on changes in solubility and activity, which are more directly related to changes in the chemical potentials, and develop methodologies that allow us to track these changes for all solution constituents.

The contribution of any component in a mixture to solute solvation is directly related to the so-called preferential interaction coefficients (PIC). Initially introduced for three-component systems of water (*w*), solute (*s*) and cosolute (*c*), PICs correspond to the excess or deficit number of cosolute molecules in the vicinity of a solute molecule relative to the composition in the bulk:

(1) $$\Gamma_{s,c}=-{\left(\frac{\partial \mu_{s}}{\partial \mu_{c}}\right)}_{T,P,m_{s}}=N_{c}-N_{w}\frac{m_{c}}{m_{w}}$$

where $\mu_{i}$ denotes the chemical potential of solution component *i*, $m_{i}$ is its bulk molality, $N_{i}$ is the number of molecules in the vicinity of the solute, T is temperature, and P is pressure [38,39,40,41,42]. Excess of a cosolute around any solute therefore translates to a stabilization (lowered free energy) of the solute compared with the same bathing solution in the absence of the cosolute [41].

We have recently extended the definition of PIC to mixtures with four or more components, while maintaining its meaning of excess number of any component *j* around another component *i*:

(2) $$\Gamma_{i,j}=-{\left(\frac{\partial \mu_{i}}{\partial \mu_{j}}\right)}_{T,P,m_{i},\mu_{k\neq i,j,r}}=N_{j}-N_{r}\frac{m_{j}}{m_{r}}$$

In Equation (2), we use i, j and k to indicate mixture components, and r replaces w in Equation (1) to indicate that any molecule in the mixture can be chosen as the *reference* component used to gauge local concentration [43].

Here, we focus on the DES formed by a 2:1 mole ratio of urea and choline chloride (CC), also named reline. We follow the solubility of β-cyclodextrin (β-CD), an excipient widely used in pharmaceutical formulations due to its capability to form host–guest complexes with hydrophobic drugs [44,45,46,47]. Among the three common and natural cyclodextrins, β-CD possesses a cavity size ideal for complex formation with many drugs, but concomitantly suffers from the lowest water solubility [48]. It has been shown that β-CD solubility in reline is enhanced by two orders of magnitude compared with its solubility in water [24,31,49]. Yet, the specific interactions that lead to this solubility enhancement remain unclear. We show that, by adding water to reline and measuring β-CD solubility in solutions with different ratios of urea and CC, it becomes possible to determine the contribution of each DES component to solvation.

We find that at almost all hydration levels (quantified in terms of water wt%), adding either urea or CC to a hydrated reline solution increases β-CD solubility. The best solubility is, therefore, attained in solutions where one of the DES components is in excess with respect to the eutectic ratio. By contrast, we have previously shown that adding CC to binary aqueous urea solutions decreases β-CD solubility. This emphasizes that solvation enhancement by each DES component sensitively depends on solution composition. We present and apply a new formulation to analytically describe and resolve the activity (or chemical potential) of urea and choline chloride at high concentrations, and use these to calculate the preferential interaction coefficients, as shown in Equation (2). We find that urea and the salt accumulate together in the vicinity of β-CD. Moreover, we show that the accumulation of DES components depends on their mole ratio and extent of hydration. Taken together, our findings provide a route to DES modification via concentration changes in its constituents, allowing rational tailoring of complex solvents for specific solvation needs.

## 2. Materials and Methods

### 2.1. Materials

Urea (≥98%), β-CD (≥97%), and choline chloride (≥98%) were purchased from Sigma-Aldrich, Merck, (Darmstadt, Germany) and were used without further purifications. Aqueous solutions were prepared using purified water (Barnstead MicroPure, Lake Balboa, CA, USA).

### 2.2. Solution Preparation

Choline chloride was dehydrated by heating to 80 °C for at least 24 h and then allowed to cool to room temperature in a vacuumed desiccator before use. Hydrated urea and CC solutions were prepared gravimetrically by mixing urea and dried choline chloride at the specified mole:mole ratio and then adding water to yield the desired hydration. This simple procedure allowed us to simultaneously set the hydration level (that we report throughout in terms of water wt%) and the ratio between DES components (that we report in terms of the urea:choline chloride mole ratio). Solutions prepared for solubility measurements spanned a wide concentration range of $m_{u}\leq 35\;\mathrm{mol}/\mathrm{kg}\;H_{2}O=35\;\mathrm{molal}$ and $m_{cc}\leq 26\;\mathrm{molal}\;$, corresponding to hydration levels at or above ~20 wt%.

### 2.3. Solubility Measurements of β-CD

Solubility measurements followed a previously described methodology [24,43]. In short, aqueous urea and choline chloride solutions were prepared gravimetrically. These solutions were added to glass vials containing an excess amount of β-CD. The solutions were stirred using a magnetic stirrer for at least 2 h and left in an oven overnight at 23.5 ± 0.5 °C. After at least 12 h, the vials were stirred again for at least 2 h and then centrifuged at 3000 rpm (4470× *g*) for at least 3 min using an IEC Centra CL2 centrifuge. The supernatant was filtered through a 0.45µm Sartorius Minisart Syringe Filter, and the optical rotational activity of β-CD was measured by a Rudolph Autopol I automatic polarimeter. The β-CD concentration was then calculated from a standard curve of aqueous solutions prepared with known concentrations (see calibration curve in Section S1 in the Supporting Information (SI)).

### 2.4. Osmotic Pressure and Water Activity Measurements

Water activity measurements were carried out using an AquaLab 4TE Dew point Water Activity Meter at a set temperature of 25 °C. The reported water activities are the average of at least 2 repeats within the instrumental error (±0.003). For water activities approaching unity, $a_{1}\to 1$ (i.e., low osmotic pressure, Π<1.5 Osmolal) complementary osmotic pressure measurements were performed using a Wescor VAPRO 5520 vapor pressure osmometer at ambient room temperature (ca. 23 °C). The measured osmolality, Π, was then converted to units of water activity, $a_{1}=\exp\left(-{\bar{V}}_{1}\Pi \right)$, where ${\bar{V}}_{1}≅0.018\;L/\mathrm{mol}$ is the partial molar volume of water at 25 °C. The resulting water activities are in Tables S6–S8 and S10 of the SI, and the procedure for deriving activities of DES components is presented in SI Sections S3 and S4.

## 3. Results and Discussion

### 3.1. β-CD Solubility

**Figure 1:**
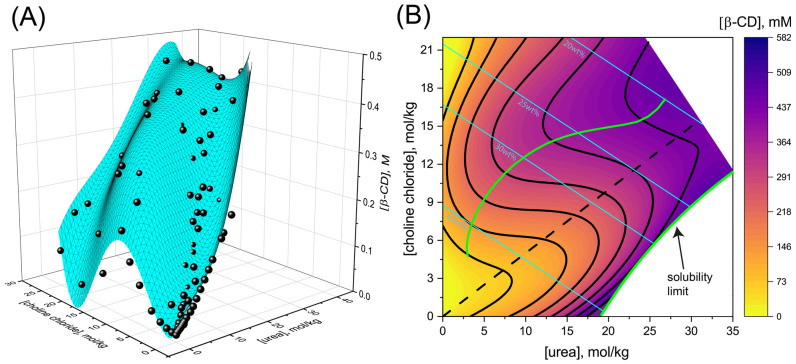
β-CD solubility versus urea and choline chloride concentrations. (**A**) Circles are data points, and the cyan surface represents a fit to Equation (S6). (**B**) Contour plot of the surface in panel (**A**). Cyan lines correspond to 20, 25, 30 and 45 wt% water. The black dashed line corresponds to the DES (reline) urea to CC ratio of 2:1. The green lines correspond to the maximum β-CD solubility for every given hydration, both above and below the 2:1 ratio. The lower green line coincides with the DES solubility limit.

Aqueous solutions of DES formed from urea and CC were prepared as described in the Materials and Methods Section 2.2. Solutions that were prepared with high urea and low CC concentrations phase separated into a liquid solution coexisting with precipitated DES (see solubility limit line in Figure 1B). At higher CC concentrations, no precipitation was observed, even well beyond urea’s solubility limit in water (~18 mol/kg). This demonstrates that CC enhances urea solubility, in line with reported phase diagrams of urea and CC mixtures [6].

We next proceeded to measure β-CD solubility in urea and CC solutions (as described in Materials and Methods Section 2.3). Figure 1A shows β-CD solubility in the hydrated DES, and Figure 1B presents the same data in terms of a contour plot of β-CD solubility generated from a two-dimensional fit to the experimental points in Figure 1A (see SI Section S2 for fit details).

Figure 1 demonstrates that both urea and choline chloride increase β-CD solubility compared with its solubility in water (~15 mM). The highest solubility is observed in solutions with the lowest hydration measured. This result is in line with previous reports, indicating that β-CD solubility in reline strongly increases with dehydration [24,31,49]. However, at any given hydration level (see cyan lines, Figure 1B), the highest β-CD solubility is observed away from the dry reline ratio (dashed line, Figure 1B). At each hydration level, the two green lines in Figure 1B indicate the urea to CC ratios that lead to the highest β-CD solubility. For example, at 30 wt% hydration, the highest solubility is observed at a urea to CC ratio close to 3:4 (~0.32 M of β-CD when CC is in excess; upper green line), as well as at a ratio of ~4:1 (~0.45 M of β-CD when urea is in excess; lower green line). At the same hydration, β-CD solubility is only ~0.25 M for the 2:1 reline composition, indicating an almost two-fold decrease in β-CD solubility.

We can, therefore, conclude that the reline ratio is not the optimal composition for β-CD solvation. In fact, the eutectic 2:1 urea to CC ratio is very close to the point of worst β-CD solvation for a given hydration. This can be explained by the fact that at this ratio, urea and CC interact most favorably with each other, as reflected by the lowest achievable melting point at the eutectic composition. Because DES components interact strongly with each other, a weaker interaction can be expected with the added β-CD solute. Finally, this is reflected by the lower β-CD solubility at the eutectic ratio.

We next use changes in solvation free energy to determine the accumulation of each DES component around β-CD. This preferential accumulation is related to the chemical potentials of each DES component, as described in Equation (2). In the following, we detail how water activity measurements, combined with solubility measurements, allow us to evaluate the chemical potential of all components in solution. In Section 3.2, we first provide a brief outline of the standard methodology to derive component activities that are applicable to the low concentration regime [50]. We then use a new approach to extend this methodology also to the very high concentration regime. Once the chemical potentials are resolved, in Section 3.3, we evaluate the PICs using Equation (2) to determine the molecular mechanism of solvation.

### 3.2. Determining Chemical Potentials

We have recently shown that the chemical potential of dilute β-CD, $\mu_{CD}$, can be derived by solubility measurements using $d\mu_{CD}=d\Delta {\bar{G}}_{CD}^{0}=-RTd\ln C_{CD}^{sat}$, where $d\Delta {\bar{G}}_{CD}^{0}=-RTd\ln C_{CD}^{sat}$ is β-CD’s solvation free energy, and $C_{CD}^{sat}$ is β-CD’s molar saturation concentration (see reference [43] and SI Section S2 for the derivation). Therefore, the derivatives of β-CD’s chemical potential that are required for PIC evaluation, Equation (2), can be calculated by fitting β-CD’s solvation free energy as a function of the component concentrations (for $\Delta {\bar{G}}_{CD}^{0}$ values and fit details, see SI Section S2 and Figure S2).

Because urea and CC are not dilute, their chemical potentials are, instead, determined using water activity measurements (see Materials and Methods Section 2.3), and are presented in terms of expansions in their concentrations:

(3) $$\mu_{cc}^{}=\mu_{cc}^{0}\left(m_{cc}\right)+\sum_{p,q}\frac{p+1}{p+q+1}A_{p,q}m_{u}^{q+1}m_{cc}^{p}$$

(4) $$\mu_{u}^{}=\mu_{u}^{0}\left(m_{u}\right)+\sum_{p,q}\frac{q+1}{p+q+1}A_{p,q}m_{u}^{q}m_{cc}^{p+1}$$

In Equations (3) and (4), $A_{p,q}$’s are fit parameters determined as detailed in the SI Section S3, p,q≥0 are integers, and $\mu_{cc}^{0}\left(m_{cc}\right)$ and $\mu_{u}^{0}\left(m_{u}\right)$ are the chemical potentials of urea and CC, respectively, in the aqueous single-DES component (binary) solutions.

To track changes in the chemical potentials using Equations (3) and (4) also requires the evaluation of changes in $\mu_{cc}^{0}\left(m_{cc}\right)$ and $\mu_{u}^{0}\left(m_{u}\right)$, as prescribed by Robinson and Stokes [51]. However, this method is impractical at high concentrations because of the limited aqueous solubility of the DES components in water, most notably of urea. Because the method inherently uses the binary single-DES component solutions as a reference (standard) chemical potential, it can only be applied to solutions that are below the solubility limit (i.e., urea concentrations lower than ~18 mol/kg at room temperature). However, we show that for higher concentrations, an alternate reference state of an aqueous mixture of both components can be used instead. Importantly, we choose the concentrations of this reference solution to be lower than the aqueous solubility of both components. This allows us to use Equations (3) and (4), combined with integration along a specific path for each component’s chemical potential differential, so that the chemical potentials can be determined at any other composition (see SI Section S4 for the derivation).

Using this extended methodology, we proceed with the derivation of the DES components’ chemical potential that uses a two-variable fit to water chemical potential in terms of component concentrations:

(5) $$\mu_{w}\left(m_{cc},m_{u}\right)=RT\ln a_{w}=\sum_{p,q}J_{p,q}m_{u}^{q}m_{cc}^{p}$$

where $J_{p,q}$ are fit parameters and p,q≥0 are integers (see SI Table S11 for values of the fit parameters). Combining these fit parameters with Equations (3) and (4), the chemical potentials of urea and CC at any concentration are (see SI Section S4 for derivation):

(6) $$\begin{array}{l} \mu_{cc}=\mu_{cc}^{0}\left(m_{cc}^{}=zm_{u}^{ref}\right)-\mu_{cc}^{0}\left(m_{cc}^{ref}\right)+\sum_{p,q}\frac{p+1}{p+q+1}A_{p,q}m_{u}^{ref,q+1}\left(z^{p}m_{u}^{ref,p}-m_{cc}^{ref,p}\right)\\ \;\;\;\;\;\;\;\;-m_{w}J_{1,0}\ln\frac{m_{u}}{m_{u}^{ref}}-m_{w}\sum_{\begin{array}{l} \;\;\;\;\;\;\;\;p,q\\ \;\;\;\;\;\;\;p\neq 0\\ \left(p,q\right)\neq \left(1,0\right) \end{array}}\frac{p}{p+q-1}J_{p,q}z^{p-1}\left(m_{u}{}^{p+q-1}-m_{u}^{ref,}{}^{p+q-1}\right) \end{array}$$

(7) $$\begin{array}{l} \mu_{u}=\sum_{p,q}\frac{q+1}{p+q+1}A_{p,q}m_{u}^{ref,q}\left(z^{p+1}m_{u}^{ref,p+1}-m_{cc}^{ref,p+1}\right)\\ \;\;\;\;\;\;\;-m_{w}J_{0,1}\ln\frac{m_{u}}{m_{u}^{ref}}-m_{w}\sum_{\begin{array}{l} \;\;\;\;\;\;\;\;p,q\\ \;\;\;\;\;\;\;q\neq 0\\ \left(p,q\right)\neq \left(0,1\right) \end{array}}\frac{q}{p+q-1}J_{p,q}z^{p}\left(m_{u}{}^{p+q-1}-m_{u}^{ref,}{}^{p+q-1}\right) \end{array}$$

In Equations (6) and (7), $z\equiv m_{cc}/m_{u}$ is the ratio between the components. The reference concentrations of CC and urea in our calculations are chosen for convenience: $m_{cc}^{ref}=1\mathrm{mol}/\mathrm{kg}$ and $m_{u}^{ref}=2\mathrm{mol}/\mathrm{kg}$. In Equation (6), the difference in the standard state chemical potentials, $\mu_{cc}^{0}\left(m_{cc}^{}=am_{u}^{ref}\right)-\mu_{cc}^{0}\left(m_{cc}^{ref}\right)$, is calculated using Equation (S23) in the SI.

Once the chemical potentials of all components are known, we can use them to calculate the PICs defined in Equation (2). Specifically, the condition of constant chemical potential in the partial derivative of Equation (2) can be satisfied for urea and CC by numerically solving Equations (6) and (7) (see reference [43] for further details).

### 3.3. Preferential Interaction Coefficients

**Figure 2:**
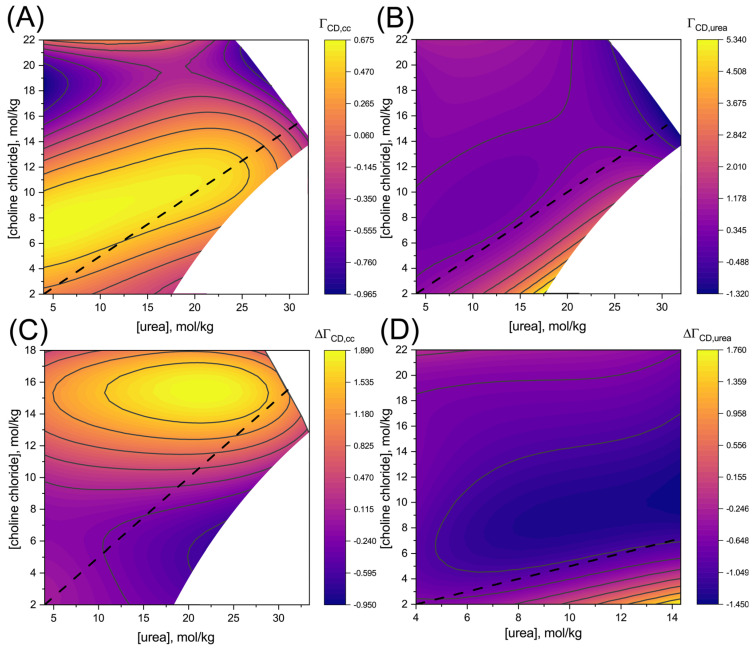
Preferential interaction coefficients of β-CD with (**A**) CC and (**B**) urea as a function of CC and urea concentration. Differences in the PICs in the mixture and their corresponding aqueous single-DES component solutions: (**C**) plot for CC, and (**D**) plot for urea (See the text for details). The black dashed lines correspond to a urea to CC ratio of 2:1.

Figure 2 shows the PICs of β-CD with CC, $\Gamma_{CD,cc}$, and with urea, $\Gamma_{CD,u}$. The positive values of the PICs indicate that both urea and CC accumulate within β-CD’s vicinity. Urea’s accumulation is strongest at low CC concentration, while it is largest for CC at ~10 mol/kg of CC. As discussed in the Introduction, PIC values are directly correlated with changes in solubility due to variations in the concentration of a solvating component, as shown in Equation (2). Indeed, high positive PIC values for urea are found at low CC concentrations (Figure 2B), where urea addition most significantly impacts β-CD solubility (Figure 1B). By contrast, larger PIC values for CC are found at higher CC concentrations (Figure 2A) where β-CD solubility increases strongly with CC addition (Figure 1B).

To determine whether accumulation of one component is increased or decreased due to the presence of the other, we compare the PICs in Figure 2A and B ($\Gamma_{CD,cc/urea}$) with those in aqueous binary solutions of CC or urea, $\Gamma_{CD,cc/urea}^{0}$ (see Figure S5 for single-DES component PICs). The difference in the preferential accumulation, $\Delta \Gamma_{CD,cc/urea}=\Gamma_{CD,cc/urea}-\Gamma_{CD,cc/urea}^{0}$, is presented in Figure 2C for CC and Figure 2D for urea. We find that at almost all hydrations and urea to CC ratios, the accumulation of urea around β-CD is lower in the mixture than when it acts alone. This may be due to the limited volume in β-CD’s vicinity, as we have also shown for other mixtures [43]. Accumulation is competitive because, in the presence of the other, the two DES components may find less accessible volume for interaction with β-CD.

Interestingly, the accumulation of CC at 10–18 mol/kg is enhanced by the presence of urea, as indicated by the positive values in $\Delta \Gamma_{CD,cc}$ (Figure 2C). We have recently reported a similar result for β-CD solubilization in aqueous mixtures of NaClO_4_ and urea [43]. Specifically, we found that repulsive (probably electrostatic) interactions limit the accumulation of ClO_4_^−^ ions in β-CD’s vicinity, but upon urea addition, these repulsions are slightly reduced, allowing more salt to accumulate. Moreover, for both salts (CC and NaClO_4_), the solubilization of β-CD by urea and the salt at lower hydration is synergistic.

For urea mixtures with CC, the solubility of urea is significantly enhanced compared with many other salts, which is another important driving force that leads to increased β-CD solubility [24,43]. By reaching much higher concentrations in dry reline, both components are able to solubilize β-CD to a higher extent. We can conclude that the remarkable solvation capability of urea with salts at low hydrations is driven by increasing each other’s solubility limit and by the urea-assisted enhancement of the salts’ accumulation.

We conclude by applying the PIC analysis to evaluate the preferential accumulation of the different DES components with each other; i.e., $\Gamma_{cc,urea}$ and $\Gamma_{urea,cc}$. We find that as hydration decreases, the accumulation of both DES components around the other increases, indicating effective attractions between the two DES components (see Figure S6 in the SI). Indeed, in line with the drastic decrease in the melting temperature of DES components upon mixing, we can expect urea and CC to interact strongly with one another [6,20]. However, at high water contents, urea becomes excluded from CC’s vicinity, as indicated by the negative $\Gamma_{cc,urea}$ values in Figure S6A. Thus, urea necessarily preferentially accumulates around water at dilute DES, but accumulates around CC at lower hydration.

## 4. Conclusions

Using solubility and water activity measurements, we have resolved the mechanism by which urea and choline chloride (CC) enhance β-CD solubility. By adding water, we have widened the stable single-phase DES region, thereby allowing us to vary the concentration ratio of urea to CC with no precipitation. This affords several advantages. First, water vapor pressure can be used to determine the chemical potential of all components in the mixture. Moreover, variations in hydrated DES composition allow us to determine the preferential interactions of different components in DES. This allows us to determine the link between DES preferential accumulation around a solute molecule and the solvation properties of the DES.

We find that for a given hydration level, the highest β-CD solubility is at a urea to CC ratio that deviates from the 2:1 eutectic ratio. Specifically, relative to the solubility at the eutectic ratio, up to a two-fold increase in β-CD solubility can be attained by either elevating or decreasing the ratio towards higher urea or CC concentrations (compare the dashed line with the two green lines in Figure 1B).

We have further developed a new methodology to interpret the chemical potential in DES solutions at low hydration. By determining the chemical potentials for a wide range of hydrated DES concentrations, we were able to evaluate preferential interaction coefficients (PICs). The PICs revealed that both urea and CC accumulate together in the vicinity of β-CD. For almost all concentrations, competition leads to a weaker accumulation of urea compared with when it acts alone in solution. By contrast, at low hydration, the presence of urea increases the accumulation of CC. Taken together, our methodology and findings provide a new way to study solvation in complex multi-component solutions, and should allow a systematic approach to modify their composition for desired solvation needs.

## Data Availability

All data is provided in the main text and Supporting Information.

## Supplementary Materials

The following supporting information can be downloaded at: https://www.mdpi.com/article/10.3390/pharmaceutics15051462/s1. Figures S1 and S2: Results and details on β-CD solubility and free energy of solvation. Figure S3: Water activity measurements. Figure S4: Details on the chemical potential evaluation of cosolutes at high concentrations. Figures S5 and S6: Preferential interaction coefficients. Tables S1–S5: Results and fitting analysis for β-CD solubility and standard free energy of solvation. Tables S6–S11: Water activity measurements and fitting analysis.
