# Supplementary material for: Deep Eutectic Solvents for Efficient Drug Solvation: Optimizing Composition and Ratio for Solubility of β-Cyclodextrin
Source: Pharmaceutics. 2023 May 11;15(5):1462. doi: 10.3390/pharmaceutics15051462 (PMC10221858; doi:10.3390/pharmaceutics15051462)
Supplement: Supplementary file 1 [file pharmaceutics-15-01462-s001.zip › pharmaceutics-2346366-supplementary.pdf]

Supporting Information

Deep Eutectic Solvents for Efficient Drug Solvation: Optimizing Composition and Ratio for Solubility of β-cyclodextrin

Ilan Shumilin, Ahmad Tanbuz and Daniel Harries[a)]

Institute of Chemistry, The Fritz Haber Research Center, and The Harvey M. Krueger Family Center for Nanoscience and Nanotechnology, Edmond J. Safra Campus, The Hebrew University, Jerusalem 9190401, Israel

[a)] Corresponding author: Daniel.Harries@mail.huji.ac.il

S1. Optical rotation of β-cyclodextrin

The solubility of β-CD in solution was determined using a calibration curve of the optical rotation, Θ , measured with known molar concentrations of β-CD, and interpolated with linear regression as shown in Figure S1.

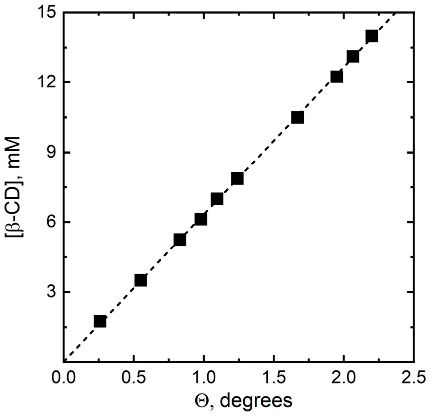

Figure S1: Optical rotation of aqueous β-CD solutions. The dashed line represents the linear fit, $[\beta\text{-CD}] = 0.00633 \times \Theta$

S2. Calculation of β-CD chemical potential using solubility measurements

Compared with water and the concentration of the DES components, β-CD is significantly dilute, so that we can use the Van' t Hoff limit, and the chemical potential of β-CD can be written as:

$$\mu_{CD}\left(m_{cc}, m_u\right) = \mu_{CD}^0\left(m_{cc}, m_u\right) + RT\ln\frac{C_{CD}}{C_{CD}^0} \quad \text{(S1)}$$

where μ_{CD}^0 is the standard chemical potential for a reference state of $C_{CD}^0 = 1mol/L$ in a solution with CC and urea concentrations of m_{CC} and m_u. This expression therefore assumes that the activity coefficient is not much different than unity. At equilibrium, for all saturated β-CD solutions the chemical potential of β-CD must be equal to its chemical potential in the precipitated (solid) state:

$$\mu_{CD}^{precipitate} = \mu_{CD}\left(m_{CC}, m_u\right) = \mu_{CD}^0\left(m_{CC}, m_u\right) + RT\ln C_{CD}^{sat} \quad \text{(S2)}$$

By differentiating Eq. (S2) with respect to the concentration of one of the DES components (as long as the solution remains saturated):

$$d\mu_{CD}^0\left(m_{CC}, m_u\right) = -RT\, d\ln C_{CD}^{sat} \quad \text{(S3)}$$

Therefore, using Eq. (S1), and differentiating Eq. (S2) at constant β-CD concentration, changes in β-CD chemical potential can be determined by:

$$d\mu_{CD}\left(m_{CC},m_u\right)=d\mu_{CD}^{0}\left(m_{CC},m_u\right)=d\Delta\bar{G}_{CD}^{0}=-RTd\ln C_{CD}^{sat} \quad \text{(S4)}$$

where $\Delta\bar{G}_{CD}^{0}\left(m_{CC},m_u\right)=-RT\ln C_{CD}^{sat}$ is β-CD's solvation free energy. Experimentally determined C_{CD}^{sat} and $\Delta\bar{G}_{CD}^{0}$ values in the single-DES component (CC or urea) aqueous solutions are in Tables S1 and S2, and values for the two-component solution are in Table S3.

Table S1: β-CD solubility and standard free energy of solvation in aqueous CC solutions.

CC molality	β-CD solubility (M)	$\Delta\bar{G}_{CD}^{0}$, J/mol
0.000	0.015	10431.886
0.796	0.016	10239.509
1.791	0.019	9842.677
3.069	0.025	9125.297
4.775	0.040	8007.056
7.162	0.081	6241.688
10.743	0.177	4293.317
9.116	0.120	5261.540
15.011	0.071	6572.203
19.977	0.024	9214.542
24.930	0.078	6320.834

Table S2: β-CD solubility and standard free energy of solvation in aqueous urea solutions.

Urea molality	β-CD solubility (M)	$\Delta\bar{G}_{CD}^{0}$, J/mol
0.000	0.015	10431.886
0.997	0.026	9076.446
0.250	0.020	9753.193
0.376	0.020	9643.354
0.500	0.022	9508.927
0.751	0.024	9253.580
4.166	0.061	6940.642
6.985	0.107	5536.929
11.119	0.180	4256.378
13.637	0.221	3738.786
1.250	0.028	8849.086
1.499	0.031	8620.437
1.752	0.034	8383.256
2.003	0.036	8213.775
2.502	0.043	7816.314
3.499	0.051	7355.208
4.999	0.074	6452.849
5.979	0.090	5958.892
7.975	0.125	5160.052
8.944	0.142	4830.782
3.499	0.051	7355.208
4.999	0.074	6452.849
5.979	0.090	5958.892
7.975	0.125	5160.052
8.944	0.142	4830.782
10.018	0.162	4512.893

Table S3: β-CD solubility and solvation free energy in aqueous mixtures of urea and CC.

[urea], mol/kg	[CC], mol/kg	β-CD solubility (M)	$\Delta\bar{G}^0_{CD}$, J/mol
0.857	0.428	0.021	9597.594
1.319	0.659	0.024	9247.322
1.930	0.965	0.028	8821.010
3.306	1.653	0.037	8158.234
5.139	2.570	0.049	7451.793
7.700	3.851	0.071	6550.039
11.573	5.786	0.127	5114.041
17.837	8.918	0.242	3518.529
31.016	15.508	0.424	2125.186
14.926	7.463	0.196	4039.837
24.413	12.206	0.343	2653.048
2.454	6.221	0.078	6320.089
12.133	6.221	0.139	4884.818
19.323	6.221	0.217	3788.197
2.762	9.830	0.232	3617.116
7.668	9.830	0.213	3831.192
8.526	2.924	0.087	6053.467
2.580	2.924	0.054	7214.535
15.776	2.924	0.170	4395.570
6.432	6.175	0.100	5706.701
2.858	13.816	0.166	4446.907
3.008	18.502	0.134	4985.178
3.371	24.474	0.157	4585.613
28.055	14.881	0.454	1959.224
22.402	11.882	0.378	2408.447
19.255	10.213	0.328	2760.078
13.357	7.085	0.182	4223.407
5.974	3.168	0.063	6837.080
4.079	2.164	0.048	7548.543
30.192	13.955	0.436	2058.575
27.708	12.807	0.423	2135.329
25.379	11.730	0.378	2408.447
21.948	10.144	0.336	2701.084
13.645	6.307	0.170	4397.447
5.269	1.790	0.061	6945.807
16.968	7.843	0.243	3505.560
17.651	10.520	0.293	3046.342
18.910	7.934	0.236	3582.904
14.492	8.540	0.229	3654.511
9.001	5.328	0.108	5523.815
15.746	6.852	0.196	4042.242
10.004	4.350	0.107	5548.565
20.084	10.042	0.301	2974.947
15.915	7.958	0.225	3700.078
19.274	19.122	0.404	2244.094
3.016	13.953	0.275	3196.024
9.813	13.635	0.408	2219.395
32.988	11.984	0.449	1984.360
26.660	17.757	0.464	1903.272
23.868	20.845	0.465	1900.231
19.927	24.831	0.326	2778.775
27.042	9.425	0.402	2260.048
19.795	14.918	0.416	2173.547
16.964	17.863	0.398	2283.187
12.942	21.720	0.273	3217.758
15.889	11.023	0.353	2582.697
12.956	16.018	0.375	2430.093
8.924	17.954	0.276	3195.170
6.938	24.614	0.169	4403.000

4.166	22.457	0.042	7834.744
4.007	20.002	0.066	6742.430
2.986	26.005	0.118	5295.089
4.974	14.817	0.286	3105.997
24.040	14.877	0.406	2233.258
22.078	17.006	0.392	2321.705
17.868	12.935	0.354	2573.820
14.849	13.899	0.338	2689.452
11.909	11.842	0.307	2926.498
11.006	9.017	0.209	3884.018
6.005	11.952	0.277	3181.828

Figure S2A shows polynomial fits that correspond to $\Delta\bar{G}_{CD}^{0}$ in the aqueous single-DES component solutions (Tables S1 and S2). The lines in Figure S2A correspond to the following polynomial fits:

$$\Delta\bar{G}_{CD}^{0}\left(m_{CC}\right)=0.003\times m_{CC}^{6}-\ 0.217\times m_{CC}^{5}+5.044\times m_{CC}^{4}+43.719\times m_{CC}^{3}+103.560\times m_{CC}^{2}$$
$$-432.035\times m_{CC}+10431.886 \qquad \text{(S5)}$$
$$\Delta\bar{G}_{CD}^{0}\left(m_{u}\right)=-1.035\times m_{u}^{3}+47.552\times m_{u}^{2}-910.662\times m_{u}+9926.914$$

β-CD solubility in aqueous urea and CC solutions (Tables S1-3) was fitted to the following 10-parameter ($p+q\leq 4$) 2D fit (Figure 1a in the manuscript):

$$m_{CD}^{sat}\left(m_{CC},m_{u}\right)=\sum_{p,q}F_{p,q}m_{u}^{q}m_{cc}^{p} \qquad \text{(S6)}$$

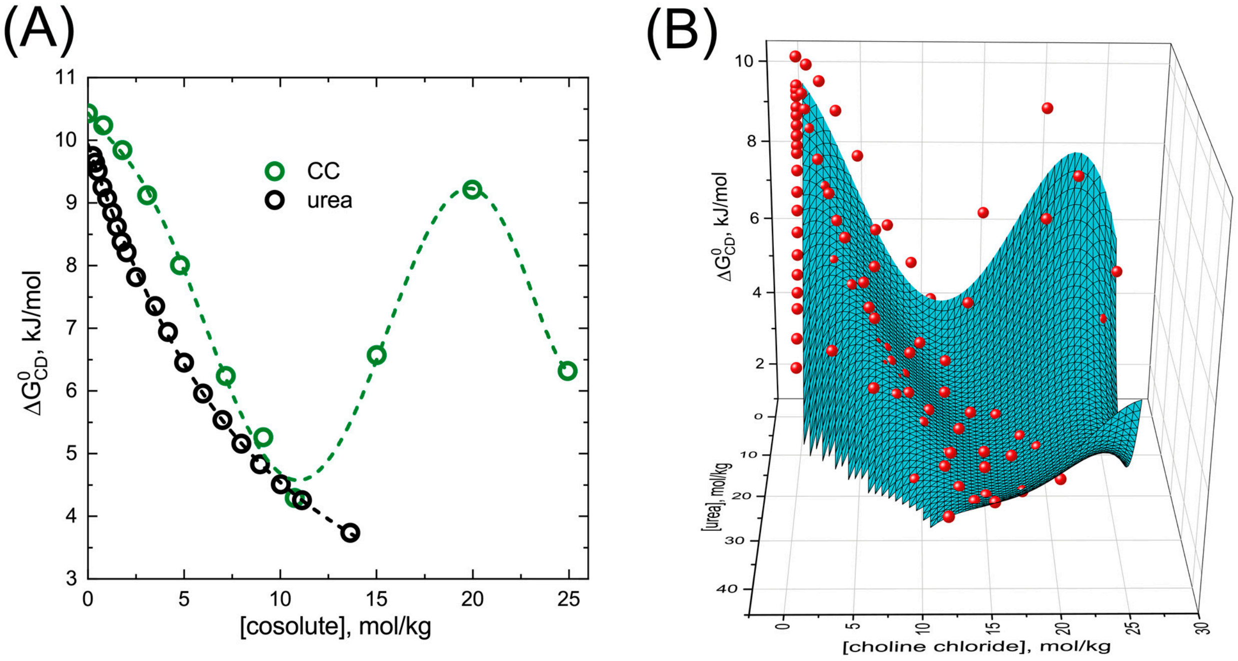

Figure S2: β-CD solvation free energy in aqueous solution of (A) single-DES component and (B) both components as a function urea and CC concentration. Dashed lines in panel (A) and cyan surface in panel (B) correspond to the fits described in the text.

and the resulting fit parameters ($F_{p,q}$'s corresponding to all $p+q \le 4$) are in Table S4.

Table S4: Polynomial fit parameters for Eq. (S6).

parameter	value
$F_{0,0}$	0.01487
$F_{0,1}$	0.02163
$F_{0,2}$	-0.00179
$F_{0,3}$	1.52E-04
$F_{0,4}$	-1.48E-06
$F_{1,0}$	-0.03399
$F_{1,1}$	-0.00113
$F_{1,2}$	-2.65E-04
$F_{1,3}$	-2.37E-06
$F_{2,0}$	0.01071
$F_{2,1}$	4.19E-04
$F_{2,2}$	1.03E-05
$F_{3,0}$	-7.96E-04
$F_{3,1}$	-1.44E-05
$F_{4,0}$	1.69E-05

β-CD solvation free energy in aqueous urea and CC solutions (Tables S1-3) was fitted to a similar 10-parameter ($p+q \le 4$) 2D fit (Figure S2b):

$$\Delta\bar{G}^{0}_{CD}\left(m_{CC}, m_u\right) = \sum_{p,q} K_{p,q} m_u^q m_{cc}^p \qquad \text{(S7)}$$

and the resulting fit parameters ($K_{p,q}$'s corresponding to all $p+q \le 4$) are in Table S5.

Table S5: Polynomial fit parameters for Eq. (S7).

parameter	value
$K_{0,0}$	10431.886
$K_{0,1}$	-640.576
$K_{0,2}$	17.910
$K_{0,3}$	-1.188
$K_{0,4}$	0.012
$K_{1,0}$	-348.798
$K_{1,1}$	106.620
$K_{1,2}$	1.413
$K_{1,3}$	0.017
$K_{2,0}$	-107.043
$K_{2,1}$	-9.769
$K_{2,2}$	-0.038
$K_{3,0}$	10.980
$K_{3,1}$	0.221
$K_{4,0}$	-0.253

S3. Calculation of CC and urea chemical potential in the low concentration regime

In a three-component solution composed of water (1), and two cosolutes (2 and 3), the Gibbs free energy differential is:

$$dG = -SdT + VdP + \mu_1 dn_1 + \mu_2 dn_2 + \mu_3 dn_3 \quad \text{(S8)}$$

Allowing to derive the following equality as a cross-differentiation relation:[1]

$$\left(\frac{\partial \mu_2}{\partial m_3}\right)_{T,P,m_2} = \left(\frac{\partial \mu_3}{\partial m_2}\right)_{T,P,m_3} \quad \text{(S9)}$$

Following the methodology developed of Robinson and Stokes,[1] we represent these derivatives by a 2-dimentional polynomial expansion in cosolute concentration of the form:

$$\left(\frac{\partial \mu_2}{\partial m_3}\right)_{T,P,m_2} = \left(\frac{\partial \mu_3}{\partial m_2}\right)_{T,P,m_3} = \sum_{p,q} \frac{(q+1)(p+1)}{p+q+1} A_{p,q} m_2^p m_3^q \quad \text{(S10)}$$

where $p, q \geq 0$ are integers, and $A_{p,q}$ are fit parameters.

Integration of Eq. (S10) yields the chemical potential of both cosolutes:

$$\begin{aligned} \mu_2 &= \mu_2^0(m_2) + \sum_{p,q} \frac{(p+1)}{p+q+1} A_{p,q} m_2^p m_3^{q+1} \\ \mu_3 &= \mu_3^0(m_3) + \sum_{p,q} \frac{(q+1)}{p+q+1} A_{p,q} m_2^{p+1} m_3^q \end{aligned} \quad \text{(S11)}$$

where μ_2^0 and μ_3^0 are the chemical potentials of both cosolutes in their respective single-cosolute solutions.

Next, we use the following set of Gibbs-Duhem equations applied to the two-cosolute solution and the single-cosolute solutions of each of the cosolutes:

$$\begin{aligned} -m_1 d\mu_1 &= m_2 d\mu_2 + m_3 d\mu_3 \\ d\mu_2^0 &= -\frac{m_1}{m_2} d\mu_{1,2} \\ d\mu_3^0 &= -\frac{m_1}{m_3} d\mu_{1,3} \end{aligned} \quad \text{(S12)}$$

where $\mu_{1,2}$ and $\mu_{1,3}$ are the chemical potentials of water in the single cosolutes solutions.

Combining the set of equations in Eq. (S12) with the cosolute chemical potentials in Eq. (S11), the following exact differential is obtained:

$$d\Delta = \left[\sum_{p,q}(q+1)A_{p,q}m_2^{p+1}m_3^q\right]dm_2 + \left[\sum_{p,q}(p+1)A_{p,q}m_2^p m_3^{q+1}\right]dm_3 \tag{S13}$$

where

$$\Delta \equiv -m_1\left(\mu_1 - \mu_{1,2} - \mu_{1,3}\right) \tag{S14}$$

Water chemical potential can be calculated from activity measurements as $\mu_1 = RT\ln a_1$.

Because the activity of water approaches unity in the limit of low cosolute concentration, $\lim_{m_2,m_3\to 0} a_1 = 1$, the following limit $\lim_{m_2,m_3\to 0}\Delta = 0$ also holds. Integration of Eq. (S13) leads to

$$\Delta = \sum_{p,q} A_{p,q}m_2^{p+1}m_3^{q+1} \tag{S15}$$

Therefore, the parameters $A_{p,q}$ that are required to evaluate changes in the cosolute chemical potential (Eq. (S11)), can therefore be extracted by fitting Δ values (that are in turn calculated using activity measurements, Eq. (S14)) to Eq. (S15). However, it is numerically easier to instead fit the function D , defined as:

$$D \equiv \frac{\Delta}{m_2 m_3} = -\frac{m_1}{m_2 m_3}\left(\mu_1 - \mu_{1,2} - \mu_{1,3}\right) = \sum_{p,q} A_{p,q}m_2^p m_3^q \tag{S16}$$

In addition, changes in the standard chemical potentials, μ_2^0 and μ_3^0, that are required in Eq. (S11), can be calculated from Eq. (S12), so that:

$$\begin{aligned}\Delta\mu_2^0 &= -m_1\int\frac{1}{m_2}d\mu_1(m_2)\\ \Delta\mu_3^0 &= -m_1\int\frac{1}{m_3}d\mu_1(m_3)\end{aligned} \tag{S17}$$

Importantly, we note that using Eq. (S17) is impossible for concentrations below the solubility limit. We therefore present a new and alternative method to calculate the chemical potentials in the following section.

In the study of DES (here, reline) in aqueous solutions, we use the notation of *cc* for component (2) and *u* for component (3). Results of water activity and chemical potential in aqueous single-DES component solutions of urea and CC are in Tables S6 and S7.

Table S6: Water activity and chemical potential in aqueous solutions of urea.

[urea], mol/kg	$a_{w,u}$ (M)	$\mu_{w,u}$, J/mol
2.000	0.968	-81.264
2.993	0.954	-116.738
4.013	0.939	-155.233
4.992	0.926	-190.853
6.032	0.912	-229.301
7.000	0.900	-260.496
7.986	0.887	-297.392
8.996	0.875	-331.160
9.973	0.864	-363.385
11.005	0.852	-397.197
11.970	0.839	-434.577
0.998	0.984	-41.230
1.200	0.981	-48.816
1.400	0.978	-56.134
1.601	0.975	-63.496
1.803	0.972	-70.680
1.997	0.969	-77.864
2.123	0.966	-84.780
0.101	0.998	-4.075
0.177	0.997	-6.723
0.253	0.996	-10.129
0.506	0.991	-21.374
0.763	0.987	-32.008
1.012	0.983	-41.899
1.271	0.979	-52.013
1.520	0.976	-61.146
1.765	0.972	-70.234
2.017	0.968	-79.560
2.513	0.962	-95.858
2.996	0.956	-110.705
2.000	0.968	-81.264
2.993	0.954	-116.738

Table S7: Water activity and chemical potential in aqueous solutions of CC.

[CC], mol/kg	$a_{w,CC}$ (M)	$\mu_{w,CC}$, J/mol
0.992	0.970	-76.785
1.993	0.941	-152.068
4.005	0.872	-338.964
5.990	0.797	-564.033
7.843	0.725	-798.902
9.731	0.656	-1047.005
14.591	0.504	-1700.005
20.180	0.379	-2407.094
23.182	0.329	-2753.215

Figure S3A shows polynomial fits corresponding to μ_w in the aqueous single-DES component solutions (Tables S6 and S7). The dashed lines in Figure S3A correspond to the following polynomial fits:

$$\mu_{w,CC} = 0.130 \times m_{CC}^3 - 5.152 \times m_{CC}^2 - 68.781 \times m_{CC}$$
$$\mu_{w,u} = 0.317 \times m_u^2 - 39.77 \times m_u \quad \text{(S18)}$$

These polynomials were then used to calculate the values of D. Table S8 shows the resulting water activity in urea and CC mixtures, along with the corresponding D values (presented in Figure S3B).

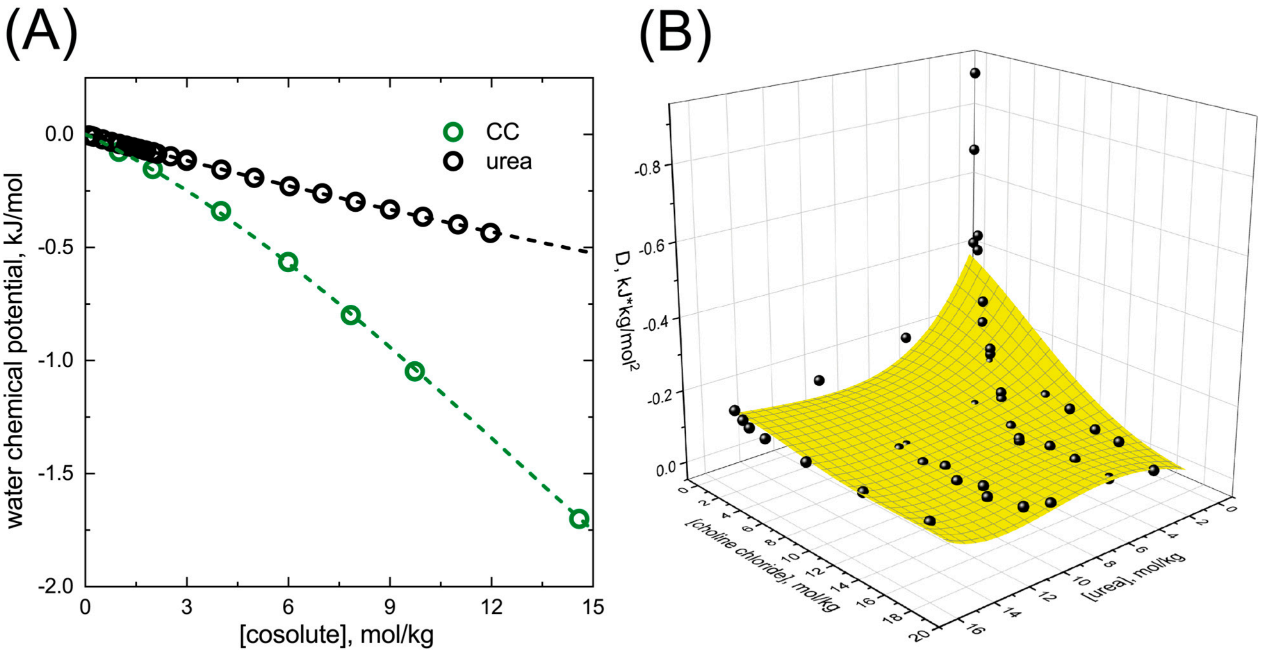

Figure S3: (A) Water chemical potential in aqueous single-DES component solutions of urea and CC as a function of concentration. Dashed lines correspond to polynomial fits (see text for details). (B) *D* as a function of urea and CC concentration. Yellow surface represents the fit to the functional form in Eq. (S16).

Table S8: Water activity and *D* at different urea and CC concentrations.

[urea], mol/kg	[CC], mol/kg	$a_{w,u}$	D, J/(mol²/kg)
11.561	5.781	0.705	-80.570
7.747	3.873	0.796	-109.788
5.136	2.568	0.865	-183.280
3.300	1.650	0.911	-239.751
1.925	0.962	0.949	-467.564
0.855	0.428	0.976	-697.793
11.514	5.757	0.708	-86.266
7.643	3.821	0.801	-126.184
5.118	2.559	0.866	-198.177
3.295	1.647	0.913	-300.696
1.920	0.960	0.948	-425.394
0.844	0.422	0.977	-911.347
17.573	8.787	0.583	-64.170
11.566	5.783	0.704	-77.237
7.694	3.847	0.797	-109.429
5.109	2.555	0.863	-159.507
3.297	1.649	0.910	-207.953
1.906	0.953	0.946	-253.245
0.855	0.428	0.976	-428.623
1.000	2.000	0.926	-184.027
4.002	2.000	0.889	-143.204
7.022	2.000	0.854	-121.664
9.025	1.999	0.833	-111.149
11.000	2.000	0.812	-102.365
12.989	1.999	0.790	-74.312
14.792	2.000	0.773	-68.812
0.998	4.999	0.825	-207.228

2.998	4.999	0.804	-120.853
4.987	4.999	0.785	-105.370
6.980	4.999	0.767	-97.560
9.935	4.999	0.740	-83.273
13.003	5.000	0.715	-76.478
14.901	5.000	0.700	-70.915
17.398	4.999	0.680	-60.748
1.999	9.925	0.639	-84.938
5.012	9.925	0.620	-77.789
7.837	9.925	0.604	-74.001
11.018	9.925	0.586	-70.041
13.987	9.925	0.571	-67.356
16.970	9.925	0.557	-64.145
17.884	2.486	0.732	-48.182
15.426	14.118	0.468	-73.154
10.987	14.648	0.471	-81.533
6.984	15.125	0.476	-101.777
3.972	15.486	0.477	-121.532
2.788	15.626	0.476	-134.374
2.301	15.684	0.476	-148.816
1.700	15.757	0.476	-167.383
1.009	10.450	0.631	-161.107
3.022	10.450	0.615	-80.739
4.875	10.450	0.604	-77.913
7.963	10.450	0.588	-76.048
9.924	10.450	0.576	-70.107
12.623	10.450	0.563	-66.890
14.949	10.450	0.551	-63.781

The fit parameters used to construct the surface fit in Figure S3B that correspond to all $A_{p,q}$ for which $p+q \leq 5$ are in Table S9.

Table S9: Polynomial fit parameters for *D*.

parameter	value
$A_{0,0}$	-425.017
$A_{0,1}$	19.562
$A_{0,2}$	2.589
$A_{0,3}$	-0.198
$A_{0,4}$	0.004
$A_{1,0}$	104.537
$A_{1,1}$	-9.281
$A_{1,2}$	0.0615
$A_{1,3}$	0.003
$A_{2,0}$	-11.783
$A_{2,1}$	0.883
$A_{2,2}$	-0.008

$A_{3,0}$	0.568
$A_{3,1}$	-0.024
$A_{4,0}$	-0.011

S4. Determining the chemical potential of each DES component in highly dehydrated solutions

In a three-component solution composed of water (1), and two cosolutes (2 and 3), the Gibbs-Duhem equation (constant T and P) is:

$$0 = m_1 d\mu_1 + m_2 d\mu_2 + m_3 d\mu_3 \quad \text{(S19)}$$

When changing solution composition for one cosolute while keeping that of the other cosolutes constant, we have:

$$\begin{aligned} \left(\frac{\partial \mu_3}{\partial m_3}\right)_{T,P,m_2} &= -\frac{m_1}{m_3}\left(\frac{\partial \mu_1}{\partial m_3}\right)_{T,P,m_2} - \frac{m_2}{m_3}\left(\frac{\partial \mu_2}{\partial m_3}\right)_{T,P,m_2} \\ \left(\frac{\partial \mu_2}{\partial m_2}\right)_{T,P,m_3} &= -\frac{m_1}{m_2}\left(\frac{\partial \mu_1}{\partial m_2}\right)_{T,P,m_3} - \frac{m_3}{m_2}\left(\frac{\partial \mu_3}{\partial m_2}\right)_{T,P,m_3} \end{aligned} \quad \text{(S20)}$$

From Eqs. (S20) and (S9), the differentials of μ_2 and μ_3 are:

$$d\mu_2 = \left(\frac{\partial \mu_2}{\partial m_3}\right)_{T,P,m_2} dm_3 + \left(\frac{\partial \mu_2}{\partial m_2}\right)_{T,P,m_3} dm_2 =$$
$$\left(\frac{\partial \mu_3}{\partial m_2}\right)_{T,P,m_3} dm_3 - \frac{m_1}{m_2}\left(\frac{\partial \mu_1}{\partial m_2}\right)_{T,P,m_3} dm_2 - \frac{m_3}{m_2}\left(\frac{\partial \mu_3}{\partial m_2}\right)_{T,P,m_3} dm_2 \qquad \text{(S21)}$$
$$d\mu_3 = \left(\frac{\partial \mu_3}{\partial m_3}\right)_{T,P,m_2} dm_3 + \left(\frac{\partial \mu_3}{\partial m_2}\right)_{T,P,m_3} dm_2 =$$
$$\left(\frac{\partial \mu_2}{\partial m_3}\right)_{T,P,m_2} dm_2 - \frac{m_1}{m_3}\left(\frac{\partial \mu_1}{\partial m_3}\right)_{T,P,m_2} dm_3 - \frac{m_2}{m_3}\left(\frac{\partial \mu_2}{\partial m_3}\right)_{T,P,m_2} dm_3$$

As described in the previous section, integration of Eq. (S21) is required in order to determine values of the chemical potential of cosolutes. However, although any arbitrarily reference states and path integrals can be chosen, only a few result in simple analytical expressions, and even fewer are compatible with the experimental analysis. For example, the method described by Robinson and Stokes is inapplicable to calculate changes in chemical potential of cosolutes in mixtures beyond their aqueous solubility (see Eq. (S17)). Because it is impossible to directly measure changes in cosolute chemical potentials, choosing a different path integral can be challenging as long as the partial derivatives in Eq. (S21) are unresolved. However, we show that a carefully thought-out path integral allows to calculate

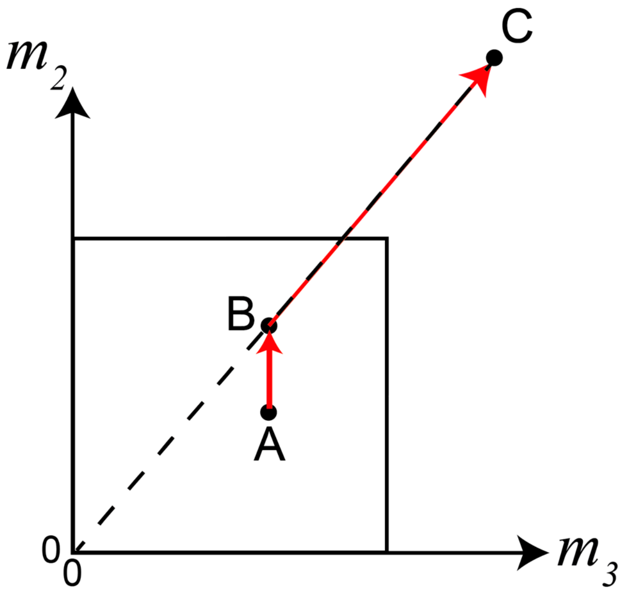

Figure S4: Schematic representation of the path integral required for cosolute chemical potential determination in the high concentration regime (see text for details). The square region corresponds to the region where both cosolutes are fully miscible in water.

the chemical potentials even without the evaluation of these partial derivatives. The process is detailed in the following.

Say we are interested in determining the chemical potential of cosolute 2 with concentrations m_2^C and cosolute 3 with concentration m_3^C so that the concentration ratio is $z = m_2^C / m_3^C$ (point C in Figure S4). The starting point of the path integral is an arbitrarily chosen point with cosolute concentrations of m_2^A and m_3^A (point A in Figure S4), which is used to define the standard chemical potential. Importantly, the concentrations of A must be sufficiently low, so that it is included within the concentration range used in the preceding chemical potential analysis (SI section S3).

The path integral is now divided into two parts:

In step one ($A \to B$, Figure S4) m_2 is varied from m_2^A to $m_2^B = m_3^B m_2^C / m_3^C = zm_3^B$, while keeping $m_2 = m_2^A = m_2^B$ constant. As long as the concentrations of point "B" are also within the low concentration regime, changes in the chemical potentials can be calculated using the set of equations (S11), so that

$$\begin{aligned} \mu_2^B - \mu_2^A &= \mu_2^0\left(m_2^B = zm_3^A\right) - \mu_2^0\left(m_2^A\right) + \sum_{p,q} \frac{p+1}{p+q+1} A_{p,q} m_3^{A,q+1}\left(zm_3^A - m_2^A\right) \\ \mu_3^B - \mu_3^A &= \sum_{p,q} \frac{q+1}{p+q+1} A_{p,q} m_3^{A,q}\left(z^{p+1} m_3^{A,p+1} - m_2^{A,p+1}\right) \end{aligned} \tag{S22}$$

where changes in the chemical potential in the single cosolute solutions are calculated using Eq. (S17):

$$\mu_2^0\left(m_2^B = zm_3^A\right) - \mu_2^0\left(m_2^A\right) = -m_1 \int_{m_2^A}^{m_2^B} \frac{1}{m_2} d\mu_1\left(m_2\right) \tag{S23}$$

The second step ($B \to C$, Figure S4) is a linear path defined by $m_3 = zm_2$, so that $dm_3 = zdm_2$. The equations in (S21) then reduces to:

$$\begin{aligned} d\mu_2 &= -\frac{m_1}{m_2}\left(\frac{\partial \mu_1}{\partial m_2}\right)_{T,P,m_3} dm_2 \\ d\mu_3 &= -\frac{m_1}{m_3}\left(\frac{\partial \mu_1}{\partial m_3}\right)_{T,P,m_2} dm_3 \end{aligned} \tag{S24}$$

Eq. (S24) can be easily integrated, as the partial derivatives can be evaluated using water activity measurements. Table S10 shows water activity measurements and water chemical potential at varying urea and CC concentrations.

Table S10: Water activity and chemical potential at varying urea and CC concentrations.

[urea], mol/kg	[CC], mol/kg	a_w	μ_w, J/mol
2.000	0.000	0.968	-81.259
2.994	0.000	0.954	-116.732
4.013	0.000	0.939	-155.225
4.993	0.000	0.926	-190.842
6.032	0.000	0.912	-229.289
7.000	0.000	0.900	-260.481
7.986	0.000	0.887	-297.376
8.996	0.000	0.875	-331.142
9.974	0.000	0.864	-363.364
11.005	0.000	0.852	-397.175
11.970	0.000	0.839	-434.552
0.997	0.000	0.984	-41.216
1.201	0.000	0.981	-48.814
1.400	0.000	0.978	-56.132
1.601	0.000	0.975	-63.496
1.803	0.000	0.972	-70.678
1.997	0.000	0.969	-77.855
2.123	0.000	0.966	-84.771
0.101	0.000	0.998	-4.069
0.177	0.000	0.997	-6.727

0.253	0.000	0.996	-10.134
0.506	0.000	0.991	-21.360
0.763	0.000	0.987	-32.009
1.012	0.000	0.983	-41.897
1.271	0.000	0.979	-52.002
1.520	0.000	0.976	-61.132
1.765	0.000	0.972	-70.219
2.017	0.000	0.968	-79.544
2.513	0.000	0.962	-95.851
2.997	0.000	0.956	-110.711
23.145	11.572	0.503	-1701.880
18.021	9.011	0.577	-1364.854
11.561	5.781	0.705	-865.084
7.747	3.873	0.796	-565.869
5.136	2.568	0.865	-360.783
3.300	1.650	0.911	-231.193
1.925	0.962	0.949	-130.620
0.855	0.428	0.976	-59.532
0.406	0.203	0.990	-24.287
69.302	34.651	0.209	-3886.333
30.665	15.332	0.410	-2208.599
18.025	9.012	0.578	-1360.344
11.514	5.757	0.708	-854.809
7.643	3.821	0.801	-549.881
5.118	2.559	0.866	-356.056
3.295	1.647	0.913	-224.888
1.920	0.960	0.948	-131.717
0.844	0.422	0.977	-57.425
69.250	34.624	0.209	-3881.226
30.608	15.304	0.409	-2216.346
17.573	8.787	0.583	-1336.514
11.566	5.783	0.704	-869.410
7.694	3.847	0.797	-562.352
5.109	2.555	0.863	-364.570
3.297	1.649	0.910	-234.133
1.906	0.953	0.946	-136.479
0.855	0.428	0.976	-61.284
145.092	72.544	0.120	-5259.885
119.805	59.902	0.138	-4911.102
101.696	50.848	0.158	-4581.203
88.094	44.047	0.183	-4206.994
77.496	38.747	0.204	-3945.649
69.013	34.506	0.230	-3644.462
62.065	31.032	0.246	-3473.636
56.271	28.135	0.262	-3322.997
51.366	25.683	0.283	-3126.408
47.160	23.579	0.304	-2949.561
43.513	21.757	0.323	-2802.088
40.320	20.160	0.345	-2634.755
37.501	18.751	0.361	-2528.361
34.996	17.499	0.384	-2370.573
32.754	16.377	0.406	-2236.551
30.736	15.368	0.428	-2106.213
1.000	2.000	0.926	-189.959
4.002	2.000	0.889	-290.538
7.022	2.000	0.854	-389.970
9.025	1.999	0.833	-454.035
11.000	2.000	0.812	-515.616
12.989	1.999	0.790	-585.349
14.792	2.000	0.773	-639.295
18.024	1.999	0.741	-742.703
18.348	2.000	0.738	-754.101
0.998	4.999	0.825	-477.156
2.998	4.999	0.804	-540.153
4.987	4.999	0.785	-599.515
6.980	4.999	0.767	-657.229
9.935	4.999	0.740	-745.715
13.003	5.000	0.715	-830.363
14.901	5.000	0.700	-883.425
17.398	4.999	0.680	-957.081
18.699	5.000	0.670	-993.081
5.005	19.787	0.376	-2423.708
11.953	19.787	0.366	-2494.226
18.400	19.786	0.356	-2561.230
25.740	19.787	0.345	-2634.755
32.964	19.786	0.335	-2709.788
36.318	19.787	0.332	-2732.823
44.314	19.787	0.321	-2815.559
1.999	9.925	0.639	-1111.111
5.012	9.925	0.620	-1184.964
7.837	9.925	0.604	-1251.827
11.018	9.925	0.586	-1324.980

13.987	9.925	0.571	-1389.046
16.970	9.925	0.557	-1452.138
19.935	9.925	0.543	-1513.681
21.844	9.925	0.534	-1553.718
26.790	9.925	0.514	-1652.146
0.000	9.925	0.653	-1057.178
66.897	32.689	0.216	-3802.178
62.948	30.157	0.231	-3630.162
56.400	25.956	0.261	-3325.366
51.929	23.087	0.288	-3085.621
43.001	17.360	0.356	-2561.230
36.316	13.071	0.427	-2109.404
31.033	9.682	0.502	-1710.267
24.980	5.798	0.608	-1233.004
20.765	3.094	0.695	-901.902
18.534	1.662	0.742	-739.027
72.094	36.024	0.198	-4014.418
149.912	74.967	0.103	-5624.813
125.936	61.544	0.125	-5164.495
109.144	52.144	0.143	-4815.887
86.635	39.542	0.182	-4230.104
81.111	36.450	0.197	-4028.228
74.840	32.940	0.216	-3804.478
58.616	23.857	0.282	-3142.208
36.673	11.573	0.455	-1953.601
27.578	6.481	0.584	-1331.546
21.441	3.045	0.691	-916.927
19.199	1.791	0.735	-764.878
16.001	0.000	0.795	-568.050
831.636	415.870	0.050	-7415.983
394.787	193.131	0.054	-7248.915
241.004	114.722	0.080	-6273.250
143.846	65.185	0.133	-5000.785
103.314	44.520	0.180	-4252.053
69.268	27.160	0.285	-3109.839
46.046	15.320	0.386	-2358.990
35.115	9.746	0.498	-1726.131
28.880	6.567	0.577	-1361.846
22.920	3.528	0.672	-987.168
19.724	1.898	0.731	-777.735
16.001	0.000	0.792	-578.045
150.701	75.259	0.122	-5222.917
111.961	60.024	0.147	-4752.696
87.735	50.496	0.169	-4406.985
57.027	38.418	0.209	-3880.396
40.395	31.876	0.254	-3400.933
30.066	27.814	0.278	-3169.657
20.217	23.941	0.324	-2794.423
10.142	19.978	0.383	-2380.915
5.466	18.138	0.424	-2129.806
0.000	15.989	0.474	-1853.174
827.672	413.745	0.049	-7465.860
416.403	216.096	0.062	-6888.661
212.445	118.085	0.087	-6044.364
95.256	61.765	0.144	-4808.977
50.574	40.293	0.201	-3982.080
31.341	31.050	0.254	-3393.125
20.679	25.927	0.302	-2966.319
11.823	21.670	0.357	-2551.150
5.652	18.705	0.420	-2151.558
0.000	15.989	0.475	-1847.422
163.930	81.959	0.111	-5442.312
132.936	70.856	0.128	-5099.647
72.738	49.284	0.185	-4189.467
57.419	43.796	0.184	-4197.541
39.843	37.498	0.211	-3854.439
25.526	32.369	0.240	-3538.596
13.508	28.061	0.275	-3203.724
7.346	25.854	0.296	-3015.193
0.000	23.221	0.330	-2746.672
25.755	12.878	0.461	-1918.954
21.607	7.401	0.595	-1289.072
22.297	8.312	0.569	-1398.179
20.630	6.113	0.631	-1141.371
23.334	9.682	0.533	-1558.827
23.894	10.420	0.516	-1640.587
19.257	4.300	0.682	-948.708
18.851	3.762	0.698	-892.113
17.884	2.486	0.732	-771.975
16.934	1.233	0.763	-669.215
21.976	13.336	0.464	-1905.550
21.000	13.453	0.463	-1907.155

19.299	13.655	0.465	-1897.543
15.426	14.118	0.468	-1883.725
10.987	14.648	0.471	-1867.349
6.984	15.125	0.476	-1838.558
3.972	15.486	0.477	-1836.998
2.788	15.626	0.476	-1840.641
2.301	15.684	0.476	-1838.558
1.700	15.757	0.476	-1841.162
25.762	12.883	0.460	-1925.144
0.257	15.928	0.478	-1830.764
25.153	12.956	0.462	-1913.047
847.309	423.549	0.054	-7239.701
365.581	195.946	0.070	-6584.752
169.496	101.496	0.099	-5737.618
79.324	58.664	0.149	-4724.194
54.455	46.849	0.172	-4359.048
30.595	35.517	0.214	-3821.792
15.468	28.332	0.259	-3351.577
6.844	24.235	0.298	-2998.517
0.000	20.984	0.334	-2716.083
862.053	431.040	0.057	-7083.749
106.437	44.929	0.201	-3972.214
160.672	80.339	0.125	-5148.617
109.832	51.608	0.170	-4396.739
73.996	31.357	0.247	-3465.295
48.561	16.982	0.376	-2422.720
40.384	12.362	0.444	-2012.629
31.137	7.136	0.569	-1398.615
25.339	3.859	0.653	-1056.419
21.908	1.920	0.711	-846.878
18.511	0.000	0.769	-649.808
0.000	10.450	0.634	-1130.396
1.009	10.450	0.631	-1142.550
3.022	10.450	0.615	-1204.633
4.875	10.450	0.604	-1248.133
7.963	10.450	0.588	-1315.902
9.924	10.450	0.576	-1365.714
12.623	10.450	0.563	-1425.783
14.949	10.450	0.551	-1477.427
18.064	10.450	0.538	-1538.456
20.150	10.450	0.529	-1576.556

We fitted the data in Table S10 to the following polynomial expansion (similar to the one in Eq. (S16)):

$$\mu_1 = \sum_{p,q} J_{p,q} m_2^p m_3^q \qquad \text{(S25)}$$

We found that a 10-parameter fit adequately represents the data. These parameters are presented in Table S11.

Table S11: Polynomial fit parameters for water chemical potential, Eq. (S25).

parameter		value
	$J_{0,0}$	41.359

$J_{p,q}$	$J_{0,1}$	-34.245
	$J_{0,2}$	-0.186
	$J_{0,3}$	0.010
	$J_{0,4}$	0.00002
	$J_{1,0}$	-105.364
	$J_{1,1}$	2.345
	$J_{1,2}$	-0.037
	$J_{1,3}$	0.0002
	$J_{2,0}$	-1.778
	$J_{2,1}$	0.002
	$J_{2,2}$	0.001
	$J_{3,0}$	0.050
	$J_{3,1}$	0.0003
	$J_{4,0}$	0.0004

Combining Eqs. (S25), (S24), and (S22), and replacing components (1-3) with (w, cc, and u), we obtain the expressions used in the manuscript for the chemical potential of the DES components (point C).

S6. Preferential interaction coefficients

Fig S5 shows the PICs defined in Eq. 1 in the main text and calculated using Eqs. (S23), (S5) and (S18) for β-CD with urea and CC, each in its own aqueous single-DES component solution.

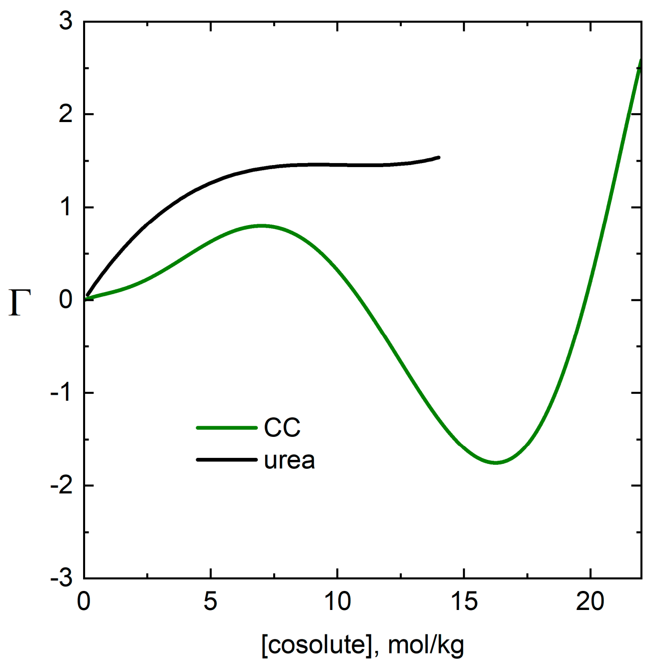

Figure S5. Single-DES component PICs of β-CD with urea (in black) and CC (in green) versus concentration.

Fig S6 shows the PICs defined in Eq. (1) in the main text and calculated using Eqs. (3) and (4) for urea and CC.

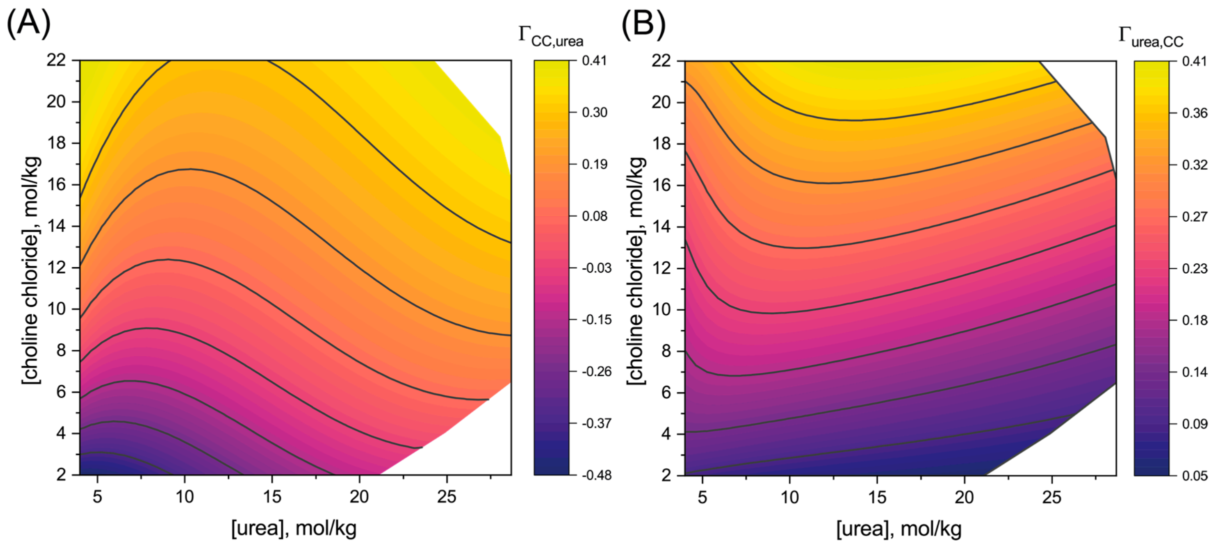

Figure S6. Contour plot of (A) CC and urea PICs, and (B) urea and CC PICs, calculated using Eqs. 6 and 7 of the main text and Eqs. (S23) and (S18).

References

[1] R.A. Robinson, R.H. Stokes, Electrolyte solutions, Courier Corporation, 2002.
